# Mitochondrial cereblon functions as a Lon-type protease

**DOI:** 10.1038/srep29986

**Published:** 2016-07-15

**Authors:** Kosuke Kataoka, China Nakamura, Toru Asahi, Naoya Sawamura

**Affiliations:** 1 Faculty of Science and Engineering, Waseda University, TWIns, 2-2 Wakamatsu, Shinjuku, Tokyo 162-8480, Japan; 2 Research Organization for Nano & Life Innovation, Waseda University, Japan.

## Abstract

Lon protease plays a major role in the protein quality control system in mammalian cell mitochondria. It is present in the mitochondrial matrix, and degrades oxidized and misfolded proteins, thereby protecting the cell from various extracellular stresses, including oxidative stress. The intellectual disability-associated and thalidomide-binding protein cereblon (CRBN) contains a large, highly conserved Lon domain. However, whether CRBN has Lon protease-like function remains unknown. Here, we determined if CRBN has a protective function against oxidative stress, similar to Lon protease. We report that CRBN partially distributes in mitochondria, suggesting it has a mitochondrial function. To specify the mitochondrial role of CRBN, we mitochondrially expressed CRBN in human neuroblastoma SH-SY5Y cells. The resulting stable SH-SY5Y cell line showed no apparent effect on the mitochondrial functions of fusion, fission, and membrane potential. However, mitochondrially expressed CRBN exhibited protease activity, and was induced by oxidative stress. In addition, stably expressed cells exhibited suppressed neuronal cell death induced by hydrogen peroxide. These results suggest that CRBN functions specifically as a Lon-type protease in mitochondria.

Cereblon (CRBN) is a gene on chromosome 3p26.2, which was identified as a causative gene for a mild type of autosomal recessive nonsyndromic intellectual disability (ID)^1^. The CRBN protein is multifunctional and immunocytochemical studies indicate that CRBN occupies multiple subcellular compartments^2^ including the nucleus, cytoplasm^3^, and endoplasmic reticulum (ER)^4^. As a cytosolic protein, CRBN is a target of thalidomide and a component of the Cullin 4-RING E3 ubiquitin ligase complex^3^. CRBN also interacts with the proteasome by binding to the β7 subunit^5^. Additionally, yeast two-hybrid screening identified CRBN as a potential binding protein of the ClC-2 channel, a voltage-gated chloride channel that mediates degradation via the ubiquitin-proteasome dependent pathway^6^,^7^. Recently, we reported that CRBN is recruited to aggresomes and functions in cytoprotection against the ubiquitin-proteasome system-impaired condition^8^. As an ER protein, CRBN is a binding partner of the large-conductance calcium-activated potassium channel, regulating surface expression of functional channels, and thereby preventing epileptogenesis^4^,^9^. As a nuclear protein, thalidomide and its analogues associate with CRBN to support proteasome-dependent degradation of the transcription factors, IKZF1 and IKZF3, which are both essential in multiple myeloma (MM). This leads to growth inhibition and apoptosis of MM cells following downregulation of c-Myc and IRF4^10^,^11^,^12^,^13^,^14^. Altogether, these reports show that CRBN functions in alteration of ubiquitination and degradation of specific targets.

CRBN contains a 237-residue Lon domain: a conserved N-terminal domain found in the Lon protease, an ATP-dependent protease within the mitochondrial matrix^1^, suggesting that CRBN may have a mitochondrial function. Mitochondria are the subcellular organelles responsible for producing intracellular reactive oxygen species (ROS), as well as generating energy in the form of ATP via oxidative phosphorylation. Because mitochondrial proteins and DNA are located in close proximity to the source of ROS, they are highly vulnerable to oxidative damage. Therefore, excessive ROS production induced by mitochondrial dysfunction can lead to mitochondrial accumulation of oxidized proteins, which contributes to mitochondrial dysfunction and cytotoxicity^15^,^16^. Accumulation of these proteins in human cells exacerbates the aging process and leads to age-related disorders such as Alzheimer’s and Parkinson’s disease^17^,^18^,^19^,^20^. To counteract oxidized protein accumulation, mammalian cells have evolved a mitochondrial protein quality control system to remove oxidized proteins^21^,^22^. In mammalian mitochondria, four main ATP-dependent proteases contribute to the mitochondrial protein quality control system^23^. Specifically, the i-AAA and m-AAA proteases are anchored within the inner membrane and expose their catalytic domains to the intermembrane space and matrix, respectively. In constrast, the ClpXP and Lon proteases are located in the matrix. Consistent with their crucial roles in protein quality control, changes in their expression levels are associated with several human diseases and the aging process^24^.

Lon protease is a highly conserved ATP-dependent serine peptidase in both prokaryotes and eukaryotes, and is required for maintenance of mitochondrial homeostasis^24^. It plays a pivotal role in mitochondrial protein quality control by degrading oxidized and misfolded proteins generated within the mitochondrial matrix^25^,^26^,^27^. Lon protease is also a stress protein that is induced by multiple stressors such as oxidative stress, heat shock, and nutrient starvation^28^. During these stresses, the *Lon* gene is up-regulated and suppresses cell death^28^. Previous studies have shown that Lon protease overexpression provides apoptotic resistance to oxidative stress in human and fungal cells^29^,^30^. By contrast, reduced levels and activities of Lon protease are associated with the aging process. In such conditions, oxidized proteins accumulate in an age-related manner and cause mitochondrial dysfunction, which ultimately leads to cell death^26^,^31^. Accumulating evidence shows that the Lon protease acts as a key antioxidant in mitochondria by limiting oxidative damage to tolerable levels.

Although CRBN contains a large, highly conserved Lon domain, the function of CRBN as a Lon protease in mitochondrial protein quality control remains unclear. Here, we show that similar to Lon protease, CRBN functions in protection against oxidative stress.

## Results

### CRBN partially localizes to mitochondria

**Figure 1:**
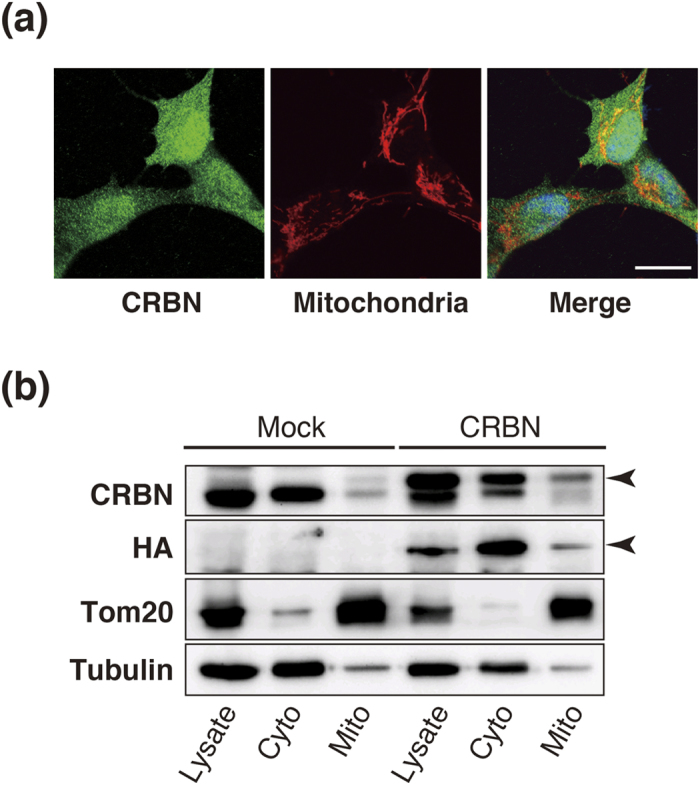
CRBN is partially distributed within mitochondria. (**a**) Confocal image analysis of SH-SY5Y cells overexpressing CRBN. CRBN is present throughout the cell including mitochondria (scale bar = 20 μm). (**b**) Western blot analysis after subcellular fractionation of SH-SY5Y cells expressing CRBN showed partial distribution of CRBN in mitochondrial fractions. α-tubulin and Tom20 were used as cytosolic and mitochondrial markers, respectively.

To examine CRBN localization in mitochondria from cultured cells, we transfected a HA-tagged CRBN expression vector^8^ into SH-SY5Y cells. Subcellular localization of exogenous CRBN was then examined by immunofluorescence microscopy. Exogenous CRBN was partially co-stained by the mitochondrial marker, MitoTracker Red (Fig. 1a), suggesting that exogenous CRBN partially localizes to mitochondria. To further characterize CRBN distribution within mitochondria, we examined the subcellular localization of exogenous CRBN using biochemical methods. In transfected SH-SY5Y cells, a CRBN protein band was detected at the predicted molecular weight (51 kDa), as previously reported^8^. Western blotting of subcellular fractions using anti-HA antibody demonstrated that CRBN was mainly present in the cytosolic fraction, but also in the mitochondrial-rich fraction (Fig. 1b).

### Mitochondrial-specific expression of CRBN in SH-SY5Y cells

**Figure 2:**
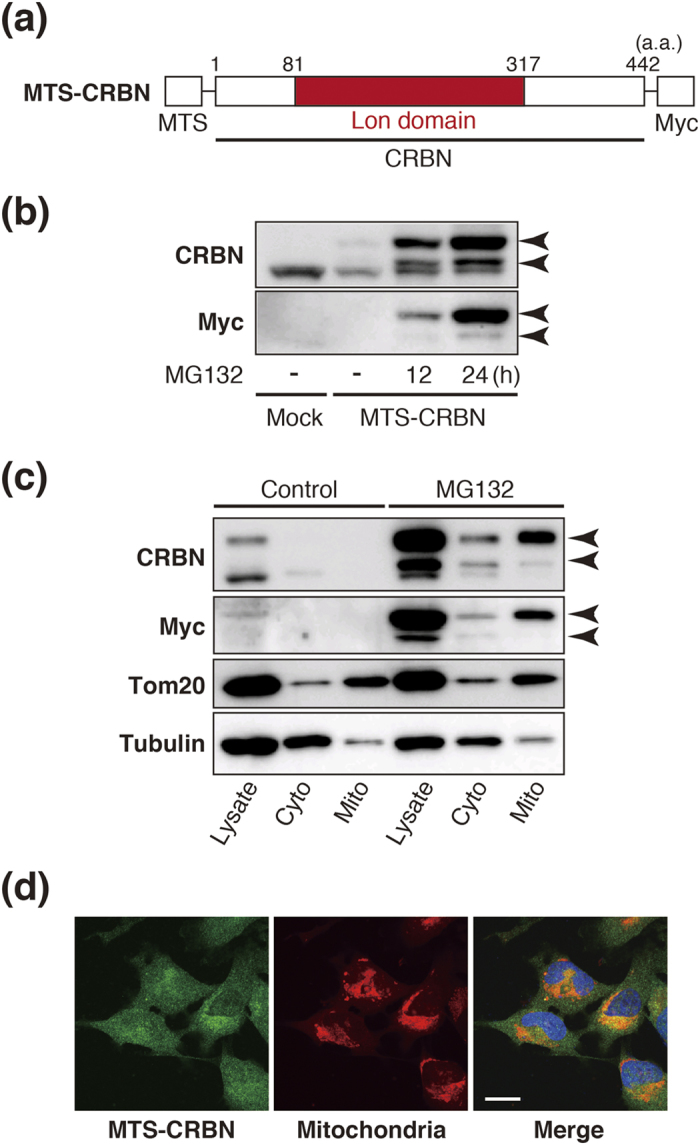
Mitochondrial-specific expression of CRBN in SH-SY5Y cells. (**a**) Schematic diagram of MTS-CRBN. CRBN contains a 237-residue Lon domain. MTS; Mitochondrial targeting signal, Myc; Myc tag. (**b**) Expression of MTS-CRBN was confirmed by western blotting. Two specific bands at molecular weights of 55 and 64 kDa were observed in MTS-CRBN stable clones, but not control SH-SY5Y cells. Expression levels of MTS-CRBN detected by both CRBN and myc-tag antibodies was enhanced by MG132 in a time-dependent manner. (**c**) Western blot analysis after subcellular fractionation of cells expressing MTS-CRBN showed that a band (indicated by the asterisk) was present in mitochondria-rich fraction of MTS-CRBN expressing SH-SY5Y cells. α-tubulin and Tom20 were used as cytosolic and mitochondrial markers, respectively. (**d**) Confocal microscopy analysis of control and MTS-CRBN-overexpressing SH-SY5Y cells. MTS-CRBN co-localized to mitochondria stained with Mito Tracker Red (scale bar = 20 μm).

To investigate the mitochondrial function of CRBN, *CRBN* cDNA was cloned into a mitochondrial targeting vector for mitochondrial expression, using a previously reported strategy^32^. The construct was transfected into SH-SY5Y cells to generate a stable cell line expressing myc-tagged mitochondrial CRBN (Fig. 2a). Western blot analysis using an anti-CRBN antibody that recognizes the N-terminal region of the protein showed that exogenously expressed CRBN was present as two bands: one with the predicted molecular weight of 55 kDa, and an additional band with a higher than expected molecular weight of 64 kDa (Fig. 2b). These bands were not detected using a myc-tag antibody. To investigate the possibility that mitochondrially-targeted CRBN (i.e., MTS-CRBN) is degraded by the ubiquitin-dependent proteasome pathway, as previously reported^8^, we performed western blot analysis after treatment with MG132, a proteasome inhibitor. Consequently, MTS-CRBN expression levels detected by both CRBN and myc-tag antibodies increased with MG132 treatment in a time-dependent manner (Fig. 2b). Next, to characterize MTS-CRBN distribution within mitochondria, we examined the subcellular localization of CRBN using biochemical methods. To characterize MTS-CRBN localization, western blotting of subcellular fractions treated with MG132 was performed, and MTS-CRBN found to specifically localize to the mitochondria-rich fraction (Fig. 2c). We also examined the subcellular localization of MTS-CRBN by immunofluorescence microscopy, and found that MTS-CRBN was co-stained with the mitochondrial marker, Tom20 (Fig. 2d).

### Mitochondrial morphology and membrane potential in MTS-CRBN overexpressed SH-SY5Y cells

**Figure 3:**
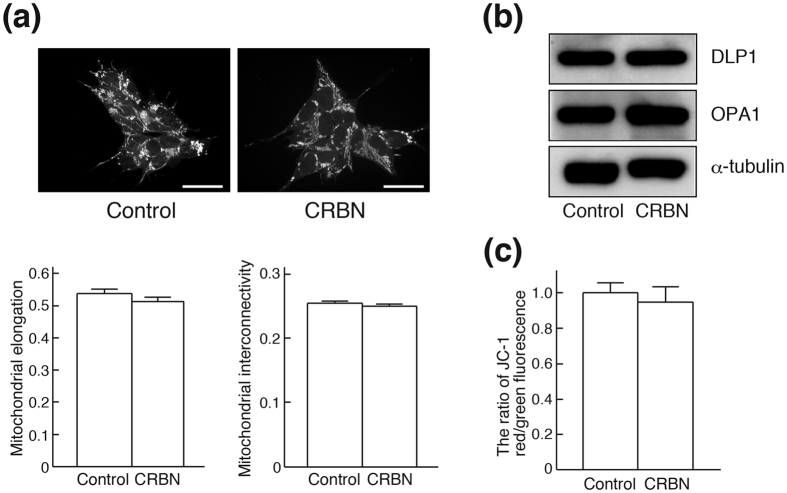
Mitochondrial morphology and membrane potential of MTS-CRBN overexpressing SH-SY5Y cells. (**a**) Three-dimensional reconstruction of Z-stacks of mitochondria labeled with MitoTracker Red CMXRos in control and MTS-CRBN-overexpressing SH-SY5Y cells (upper panel, scale bar = 30 μm). Quantitative image analysis of mitochondrial elongation and interconnectivity in control and MTS-CRBN-overexpressed SH-SY5Y cells. Mean circularity and area/perimeter ratio were used as indices of elongation and interconnectivity, respectively, as described previously^46^. There were no significant differences between control and MTS-CRBN overexpressed SH-SY5Y cells (lower panel). (**b**) Expression levels of DLP1 and OPA1, proteins associated with mitochondrial fission and fusion processes, were confirmed by western blotting. Expression levels of both proteins were similar in control and MTS-CRBN-overexpressed SH-SY5Y cells. (**c**) Mitochondrial membrane potential was measured using JC-1 reagent. Red/green fluorescence intensity ratio was determined in control and MTS-CRBN overexpressed SH-SY5Y cells after JC-1 reagent treatment. Overexpression of MTS-CRBN did not affect mitochondrial membrane potential. *N* = 6.

Regulation of mitochondrial dynamics (i.e., fusion and fission) plays a major role in quality control^33^. Fusion alleviates stress by mixing the contents of impaired mitochondria with intact ones^34^. In contrast, in fission, dysfunctional mitochondria are segregated for autophagy (mitophagy)^35^,^36^. To determine the effect of MTS-CRBN overexpression on mitochondrial dynamics, we examined mitochondrial morphology in stable MTS-CRBN overexpressed SH-SY5Y cells. Accordingly, we obtained three-dimensional projection Z-stack images of mitochondria stained with MitoTracker Red (Fig. 3a, upper panel), and examined their morphology by measuring mitochondrial elongation and interconnectivity using NIH ImageJ (Fig. 3a, lower panel). Mitochondrial elongation and interconnectivity scores were not significantly different between MTS-CRBN overexpressed and control SH-SY5Y cells (Fig. 3a). Western blot analysis of the OPA1 and DLP1 proteins, which are involved in mitochondrial fusion and fission, respectively, revealed similar expression levels of both proteins between MTS-CRBN overexpressed and control cells (Fig. 3b). These results indicate that MTS-CRBN is not associated with regulation of mitochondrial dynamics. To investigate the effect of MTS-CRBN overexpression on mitochondrial function, we measured mitochondrial membrane potential using JC-1 fluorescent dye, which exhibits a mitochondrial proton motive force (PMF)-dependent shift of fluorescence emission from green to red. Fluorescence spectrophotometer analysis showed that the ratio of red/green fluorescence intensity was not significantly altered by MTS-CRBN overexpression (Fig. 3c), suggesting that MTS-CRBN does not affect mitochondrial membrane potential. Altogether, MTS-CRBN has no apparent effect on mitochondrial functions involving mitochondrial fusion, fission, and membrane potential.

### Mitochondrial CRBN functions as a Lon-type protease

**Figure 4:**
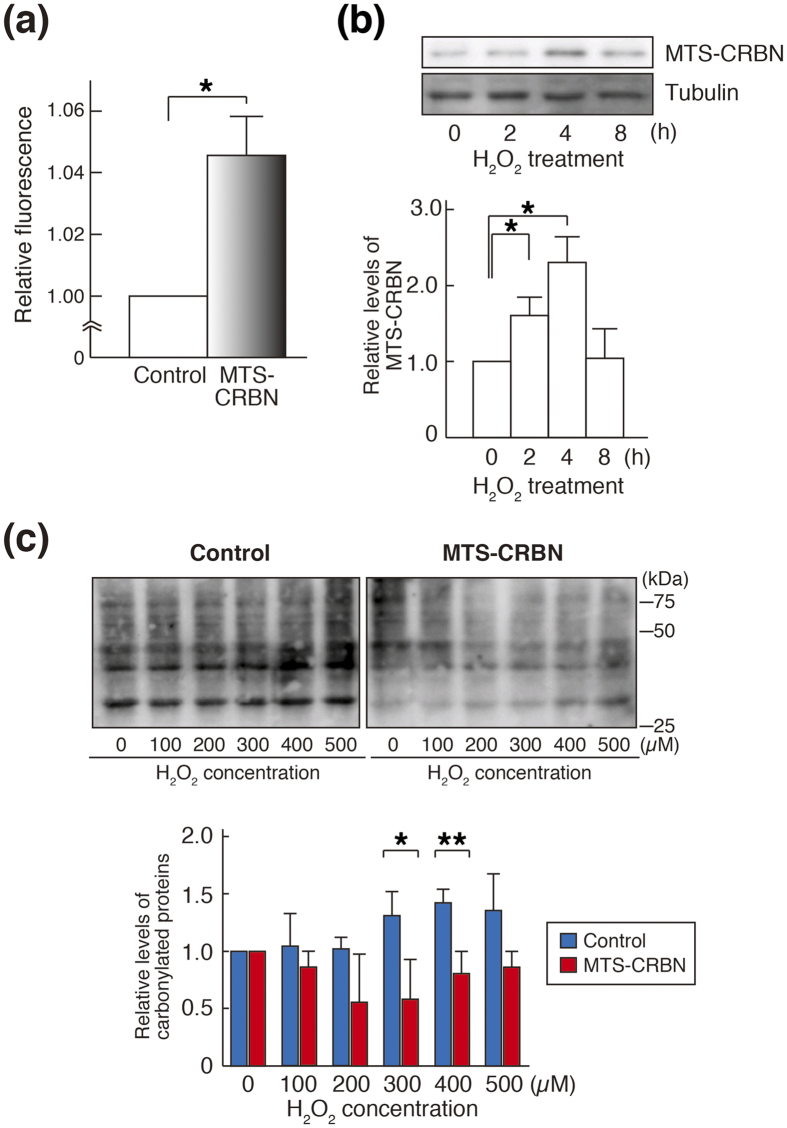
Functions of MTS-CRBN as a Lon-type protease. (**a**) Proteolytic activities of MTS-CRBN were measured using FITC-casein. The fluorescence intensity of FITC casein in the mitochondrial fraction of MTS-CRBN overexpressed cells were significantly increased compared with that of control cells (*p < 0.05). (**b**) Expression levels of MTS-CRBN under oxidative stress conditions were measured by western blotting. The level of MTS-CRBN was transiently increased by the treatment of hydrogen peroxide (*p < 0.05). (**c**) The level of carbonylated proteins, a marker of oxidative stress, was detected by western blotting using antibody against 2,4-dinitrophenol (DNP). The level of carbonylated proteins produced by oxidative stress was suppressed in total cellular extract of MTS-CRBN overexpressed SH-SY5Y cells compared with that of control cells (*p < 0.05, **p < 0.01).

We examined the proteolytic activity of mitochondrially-expressed CRBN using FITC-casein, which has been reported as a substrate for a broad spectrum of proteases, including Lon protease^31^. The proteolytic activity was significantly increased in mitochondria fraction of MTS-CRBN overexpressed SH-SY5Y cells compared to that of control cells (^*^p < 0.05, Fig. 4a). Lon protease is induced by multiple stressors, including hydrogen peroxide, and contributes to protection against these stresses^25^,^28^, we next analyzed the protein expression levels of mitochondrially-targeted CRBN under the oxidative stress condition. After treatment of hydrogen peroxide, MTS-CRBN expression was transiently increased in MTS-CRBN overexpressed cells (*p < 0.05, Fig. 4b). We also observed that endogenous CRBN and Lon were induced at the same levels in oxidative stress conditions, and the expression of Lon in SH-SY5Y cells was downregulated by increased expression of MTS-CRBN under oxidative stress (Supplementary Fig. 1). Oxidative stress is associated with the formation of oxidatively modified biomolecules, including proteins, lipids and DNA^15^,^16^,^17^. The level of carbonylated proteins, a marker of oxidative stress, was reduced in total cellular extract of MTS-CRBN overexpressed SH-SY5Y cells (*p < 0.05, Fig. 4c). The level of oxidized proteins, was also reduced in mitochondrial fraction of MTS-CRBN overexpressed SH-SY5Y cells (Supplementary Fig. 2). Altogether, these results indicate that mitochondrially-targeted CRBN exhibits Lon-type protease activities.

**Figure 5:**
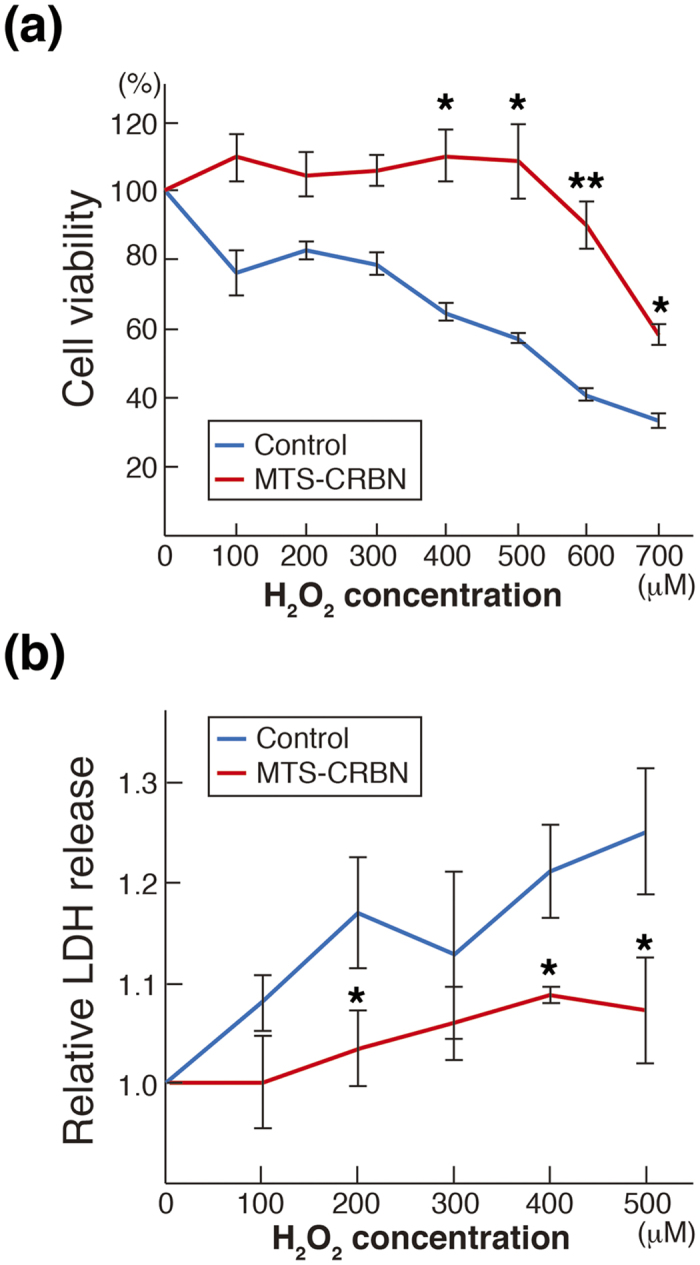
MTS-CRBN suppresses cell death induced by oxidative stress. (**a**) Cell viability after treatment with different concentrations (0–700 μM) of hydrogen peroxide was performed by measuring intracellular levels of the tetrazolium salt. SH-SY5Y cell viability decreased after hydrogen peroxide treatment in a dose-dependent manner, whereas MTS-CRBN overexpression significantly increased cell viability compared with control SH-SY5Y cells (*p < 0.05, **p < 0.01). (**b**) Cell death of MTS-CRBN overexpressed cells induced by different concentrations of hydrogen peroxide were measured using the LDH assay. Cell death of MTS-CRBN overexpressed SH-SY5Y cells induced by hydrogen peroxide was suppressed compared with control SH-SY5Y cells (*p < 0.05).

Because CRBN contains a putative Lon domain, we determined if overexpressing MTS-CRBN cells show protection against hydrogen peroxide. Cells were exposed to various concentrations of hydrogen peroxide, and cell viability determined by measuring the levels of intracellular 3-(4,5-dimethylthiazol-2-yl)-5-(3-carboxymethoxyphenyl)-2-(4-sulfophenyl)-2H-tetrazolium, which correlates with viability in cultured cells. SH-SY5Y cell viability decreased after hydrogen peroxide treatment in a dose-dependent manner (Fig. 5a), whereas MTS-CRBN overexpression significantly increased cell viability compared with control SH-SY5Y cells (*p < 0.05, **p < 0.01, Fig. 5a). Moreover, using the lactate dehydrogenase (LDH) assay, we examined cell death in MTS-CRBN overexpressed cells after hydrogen peroxide treatment. Hydrogen peroxide treatment of SH-SY5Y cells caused increased LDH release (Fig. 5b), which correlated with increased cell death. Hydrogen peroxide-induced cell death in MTS-CRBN overexpressed SH-SY5Y cells was dramatically suppressed compared with control SH-SY5Y cells (*p < 0.05, Fig. 5b). Taken together, these results indicate that MTS-CRBN suppresses cytotoxicity induced by oxidative stress.

## Discussion

In this study, we have demonstrated that CRBN partially distributes within mitochondria. CRBN contains a highly conserved, large Lon domain, which is found in the N-terminus of the ATP-dependent Lon protease that localizes to the mitochondrial matrix, and raises the possibility that CRBN might have a mitochondrial function. To focus on the mitochondrial-specific function of CRBN, we constructed an expression vector of MTS-CRBN, in which a MTS enables exogenous CRBN to target mitochondria. Using this vector, we established a stable cell line expressing MTS-CRBN, and confirmed CRBN expression in mitochondria. Expression of MTS-CRBN had no apparent effect on mitochondrial functions involving mitochondrial fusion, fission, and membrane potential. The Lon protease is reported to be induced by oxidative stress and have protective effects against extracellular stress, including oxidative stress induced by hydorgen peroxide^25^,^28^. We found that MTS-CRBN expression exhibited the mitochondrial proteolytic activity, MTS-CRBN was transiently induced by oxidative stress, and MTS-CRBN expression suppressed the formation of carbonylated proteins produced by the stress. Using the established cell line expressing MTS-CRBN, we found that MTS-CRBN has protective functions against oxidative stress. Our data suggests that, similar to the Lon protease, mitochondrial CRBN has a protective function against oxidative stress.

The mitochondrial quality control system is pivotal for cell survival. Three major ATP-dependent proteases (m-AAA, ClpXP, and Lon) can recognize abnormal proteins generated in the mitochondrial matrix and subsequently degrade them^24^,^37^. The m-AAA protease is embeded within the mitochondrial inner membrane, with its catalytic sites exposed to the mitochondrial matrix, while ClpXP and Lon proteases are active within the mitochodnrial matrix^24^,^38^. The ClpXP protease is composed of a proteolytic subunit (ClpP) and a chaperone-like subunit (ClpX) that carries a AAA+ domain^39^. Lon protease is comprised of three domains: an N-terminal domain, central ATPase domain, and a C-terminal protease domain, and ordinarily forms a homo-hexamer. The CRBN protein contains the N-terminal domain of the ATP-dependent Lon protease without Walker A and B motifs necessary for binding and hydrolysis of ATP and a peptidase domain for catalytic activity^1^,^9^, which suggests that CRBN may function as complex with another protein containing ATPase and protease domains.

Impaired maintenance of mitochondrial proteins by these proteases is associated with multiple neurodegenerative and neurodevelopmental diseases. For example, SCA28 is a form of spinocerebellar ataxia, specifically, a progressive neurodegenerative disorder characterized by poor coordination of legs, hands, speech, and eye movements. SCA28 is caused by impaired degradation of abnormal mitochondiral matrix proteins due to a mutation in *AFG3L2*, which encodes a subunit of the hexameric m-AAA protease^40^. Furthermore, disruption in the mitochondrial proteome control surveillance system caused by a mutation in *SPG7* that encodes a m-AAA subunit has been reported for an autosomal recessive form of hereditary spastic paraplegia. This is a complex form of a late-onset progressive neurodegenerative disorder which involves progressive spasticity, epilepsy, and ID^41^. In *Drosophila melanogaster*, i-AAA deletion mutants display increased ROS levels, abnormal mitochondria, and neurodegeneration^42^. Moreover, decreased Lon protease expression, at both the RNA and protein level, has been found in a patient with SPG13, one type of an autosomal dominat form of hereditary spastic paraplegia. This suggests that mitochondrial quality control, which is performed by Lon protease in the mitochondrial matrix, may be associated with pathogenesis of a wide range of neuronal diseases including neurodegenerative disorders, epilepsy, and ID^43^. Perrault Syndrome is characterized by sensorineural hearing loss and ovarian failure, and reported to be caused by mutations in CLpP, which encodes a proteolytic CLpXP subunit^44^. Recently, CODAS syndrome, a multi-system developmental disorder characterized by cerebral, ocular, dental, auricular, and skeletal anomalies, was associated with mutations of *LONP1*, encoding mitochondrial AAA^+^ Lon Protease^45^. Thus, these developmental disorders are associated with dysfunction of mitochondrial proteases. As with other mitochondrial proteases, the putative Lon domain within CRBN is functional, and a defect in its function may be of importance to ID development.

Here, we provide insight into the physiological function of CRBN in cytoprotection against oxidative stress, and show that it is similar to Lon protease. Thus, as regards Lon protease, defective CRBN function at the neurodevelopmental stage may contribute to ID pathogenesis, as seen in other neuropsychiatric diseases. It will be of interest to examine the Lon protease function of CRBN in model animals and human subjects of neurodevelopmental disorders.

## Methods

### Reagents

The following antibodies were used: mouse anti-hemagglutinin (HA) antibody (Covance, Berkley, CA, USA); mouse anti-myc antibody (Millipore, Billerica, MA, USA); rabbit anti-CRBN antibody (Sigma Aldrich, St. Louis, MO, USA); mouse anti-α-tubulin (Cell Signaling Technologies, Beverly, MA, USA); rabbit anti-Tom20 (F-10) (Santa Cruz Biotechnology, Dallas, TX, USA); mouse anti-DLP1 antibody (BD Bioscience, San Jose, CA, USA); mouse anti-OPA1 antibody (BD Bioscience); anti-rabbit IgG, HRP-linked whole antibody, donkey (GE Healthcare, Waukesha, WI, USA); anti-mouse IgG, HRP-linked whole antibody, sheep (GE Healthcare); Alexa Fluor488-labeled anti-mouse IgG (Life Technologies, Carlsbad, CA, USA); and Alexa Fluor594-labeled anti-rabbit IgG (Life Technologies); rabbit polyclonal anti-dinitrophenyl (DNP) antibody (SHIMA Laboratories, Tokyo, Japan). MG132 (Peptide Institute, Osaka, Japan) was dissolved in dimethyl sulfoxide and stored at −20 °C.

### Construction of CRBN expression vectors

*CRBN* cDNA was amplified from Genome Network Project Human cDNA Clones (IRAL038I16, Riken BioResource Center, Kanagawa, Japan). *Sal*I/*Not*I fragments containing CRBN were isolated and ligated into the mammalian expression vector, pCMV/myc/mito (Life Technologies), which was digested with the same endonucleases. Construction of the plasmid pRK5-HA-CRBN has been described previously^8^. Both recombinant plasmids were used for further studies. The entire nucleotide sequence was confirmed by DNA sequencing.

### Cell culture and transfection

The SH-SY5Y cell line, a dopaminergic human neuroblastoma cell line, was maintained in low-glucose Dulbecco’s modified Eagle’s medium (DMEM) (Wako, Osaka, Japan) containing 10% (v/v) fetal bovine serum (FBS) and 1% penicillin-streptomycin. For construction of stable cell lines, SH-SY5Y cells were transfected with pRK5-HA-CRBN and pcDNA3.1 (Life Technologies) or pCMV/myc/mito-CRBN using Lipofectamine 2000 Reagent (Life Technologies), according to the manufacturer’s instructions. Clones that survived in 1.0 mg/ml G418 were isolated and maintained in DMEM medium (Wako) supplemented with 10% FBS containing 1.0 mg/ml G418 at 37 °C in 5% CO_2_. To inhibit proteasome activity, SH-SY5Y cells were treated with 5 μM MG132 for 12 hr.

### Biochemical assays

Total protein extracts were prepared from SH-SY5Y cells as described previously^8^. Subcellular fractionation and western blotting were performed as described previously^32^. Briefly, cells were homogenized in ice-cold buffer (50 mM Tris·HCl, pH 7.4/150 mM NaCl) with protease inhibitor mix (Roche, Bazel, Switzerland) by using a motor-driven Teflon homogenizer. Homogenates containing equal amounts of protein were centrifuged at 1,000 × *g* for 10 min at 4 °C to obtain a crude nuclear pellet and postnuclear supernatant. The postnuclear supernatant was further centrifuged at 13,000 × g for 30 min at 4 °C to obtain the cytoplasmic fraction (supernatant) and crude mitochondrial fraction (pellet). The supernatant is considered to be cytoplasmic fraction with enriched in α-tubulin, and the final mitochondrial fraction is considered to be a mitochondrial fraction with enriched in the mitochondria outer membrane protein Tom20.

### Immunofluorescence microscopy

For subcellular localization of HA-CRBN, a standard protocol using MitoTracker Red (Life Technologies) and HA-tag antibody was performed, as described previously^32^. For subcellular localization of myc-CRBN with a mitochondrial targeting sequence (MTS-CRBN), cells were methanol-fixed and co-stained with MitoTracker Red and mouse anti-myc antibody. Fixed cells were incubated with Alexa488 goat anti-rabbit, and then mounted in VECTASHIELD with DAPI (Life Technologies) for imaging. Images of these samples were acquired by confocal microscopy (FV1000; Olympus, Tokyo, Japan).

### Quantification of mitochondrial morphology

Four μm thick Z-stacks of 7–8 slices for each image were acquired and three-dimensional projections generated by fluorescence microscopy (BZ-X700; Keyence, Osaka, Japan). To quantify parameters of mitochondrial morphology, the acquired images were inverted as black pixels and a threshold applied using NIH ImageJ (NIH, Bethesda, MD, USA) to optimally resolve individual mitochondria. Mitochondria were outlined using the “analyze particles” feature. Circularity and mean area/perimeter ratio were used as a measure of mitochondrial elongation and interconnectivity, respectively.

### Mitochondrial membrane potential assay

Mitochondrial membrane potential was determined using JC-1 (Life Technologies). Cells were grown in 96-well plates and incubated with JC-1 for 30 min. The fluorescence emission shift from green (529 nm) to red (590 nm) was measured, and the membrane potential represented as the red/green fluorescence intensity ratio.

### Proteolytic activities assay

Proteolytic activities were assayed, using FITC-casein (Sigma Aldrich) as a substrate, according to a previously published method^31^ with minor modifications. Crude mitochondria fractions were prepared as previously described^32^, and the mitochondrial pellet were suspended with 100 μL of buffer (50 mM Tris/HCl, pH 7.9, 10 mM MgCl_2_, 1 mM 2-mercaptoethanol, 0.05% Triton X-100). The protein concentration was determined with BCA protein assay (Thermo Fisher Scientific, Waltham, MA), and the same content of mitochondrial fractions (50–100 μg) were used for further experiment. 5 μg of FITC-casein was added to the mitochondrial suspension. Fluorescence was measured with excitation/emission wavelengths of 495/515nm.

### Cell viability assay

Cell viability was determined using the CellTiter 96^®^ Aqueous One Solution Cell Proliferation Assay kit (Promega, Madison, WI, USA), according to the manufacturer’s instructions. For the cytotoxicity assay, CytoTox 96^®^ Non-Radioactive Cytotoxicity Assay (Promega) was used, according to the manufacturer’s instructions.

### RNA extraction and quantitative reverse transcription-polymerase chain reaction (qRT-PCR)

Sepasol-RNA I Super G (Nacalai Tesque, Kyoto, Japan) and the ReverTra Ace^®^ qPCR RT Kit (TOYOBO) were used for extraction of total RNA from SH-SY5Y cells and cDNA synthesis for qRT-PCR, according to the manufacturers’ instructions, respectively. qRT-PCR experiments were performed using Thunderbird SYBR qPCR Mix (TOYOBO) on the StepOne Real Time PCR System (Applied Biosystems, Foster City, CA). The following primers were used for CRBN amplification: CRBN: 5′-CAGTCTGCCGACATCACATAC-3′ and 5′-GCACCATACTGACTTCTTGAGGG-3′. The following primers were used for human Lon protease and Actin amplification, as described previously^28^. Lon: 5′-ATGGAGGACGTCAAGAAACG-3′ and 5′-GATCTCAGCCACGTCAGTCA-3′; actin: 5′-TTGTTACAGGAAGTCCCTTGCC-3′ and 5′-ATGCTATCACCTCCCCTGTGTG-3′.

### Statistical analysis

In all experiments, data were expressed as mean ± SD from three to five independent experiments. Data were analyzed using the Student’s *t*-test, and differences between samples were considered statistically significant when *p* < 0.05.

## Additional Information

**How to cite this article**: Kataoka, K. *et al*. Mitochondrial cereblon functions as a Lon-type protease. *Sci. Rep.* **6**, 29986; doi: 10.1038/srep29986 (2016).

## Supplementary Material

Supplementary Information
