# Supplementary material for: Mitochondrial cereblon functions as a Lon-type protease
Source: Sci Rep. 2016 Jul 15;6:29986. doi: 10.1038/srep29986 (PMC4945938; doi:10.1038/srep29986)
Supplement: Supplementary Information [file srep29986-s1.pdf]

Supplementary Information

# Mitochondrial cereblon functions as a Lon-type protease

Kosuke Kataoka[1], China Nakamura[1], Toru Asahi[1,2],

and Naoya Sawamura[1,2]*

1. Faculty of Science and Engineering, Waseda University, TWIns, 2-2 Wakamatsu, Shinjuku, Tokyo 162-8480, Japan
2. Research Organization for Nano & Life Innovation, Waseda University

*Corresponding Author:

Naoya Sawamura, Ph.D.

Associate Professor

Research Organization for Nano & Life Innovation, Waseda University

#03C309, TWIns, 2-2 Wakamatsu, Shinjuku, Tokyo 162-8480, Japan.

Phone & Fax: +81-3-5369-7327

Email: naoya.sawamura@gmail.com

**Supplementary Figure 1**

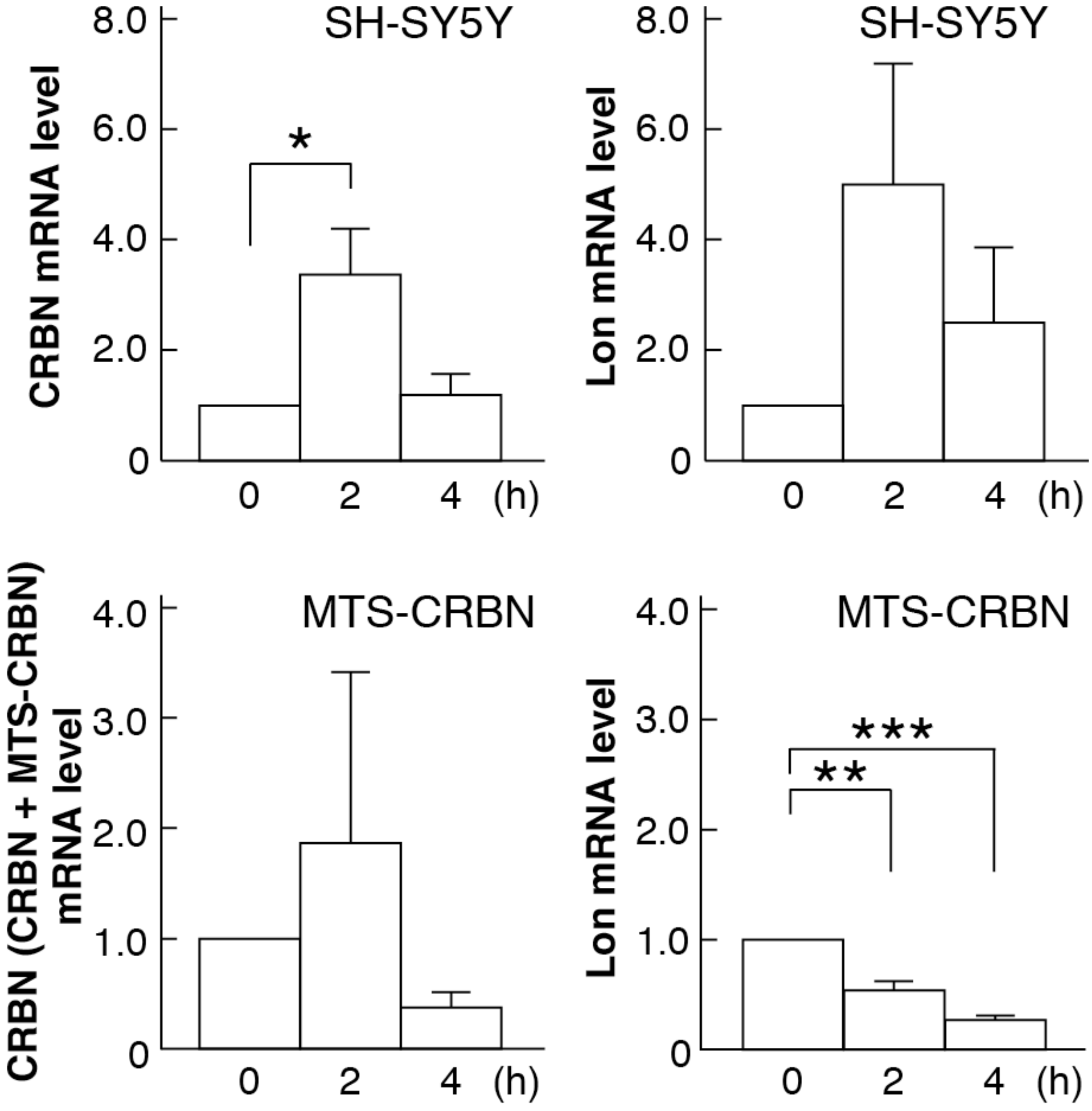


**mRNA expression of CRBN and Lon in normal and MTS-CRBN overexpressed SH-SY5Y cells under oxidative stress condition.**

To know that CRBN has a similar role to Lon protein in the response to

oxidative stress, the mRNA expression levels of *Lon* and *CRBN* were analyzed by qRT-PCR. Induction of Lon was observed at 2 h after hydrogen peroxide treatment, with the highest protein induction, approximately 5.0-fold in SH-SY5Y cells. Induction of endogenous CRBN was also observed after hydrogen peroxide treatment with the highest inductions of 3.4-fold (at 2 h) in SH-SY5Y cells (*$p < 0.05$). MTS-CRBN expression in SH-SY5Y cells decreased the expression level of *Lon* mRNA to 54% (at 2 h), and 27% (at 4 h) (**$p < 0.005$, ***$p < 0.0001$ respectively), under oxidative stress. In this condition, the level of total CRBN (which contain both endogenous CRBN and MTS-CRBN) expression in MTS-CRBN overexpressed cells was induced, with the modest induction of 1.9-fold. We have already observed that MTS-CRBN specific expression was induced in MTS-CRBN overexpressed cells, with the highest induction of 2.3-fold (at 4 h) after $H_2O_2$ treatment (*$p < 0.05$, Fig. 4B). These results suggest that endogenous CRBN and Lon were induced at the same levels in oxidative stress condition, and Lon expression in SH-SY5Y cells is downregulated by increased expression of MTS-CRBN under oxidative stress. *CRBN* and *Lon* mRNA levels were normalized to *actin* mRNA expression. The data are represented as means ± SEM (SH-SY5Y: n=4, *$p < 0.05$; MTS-CRBN: n=3, **$p < 0.005$, ***$p < 0.0001$).

**Supplementary Figure 2**

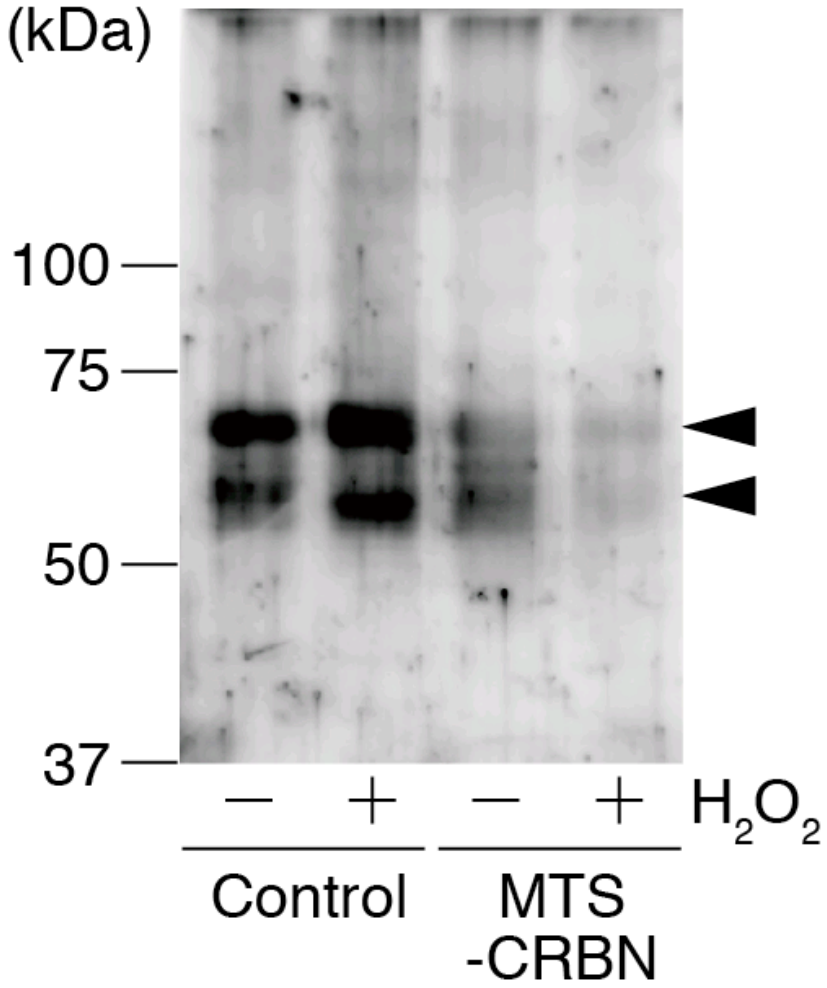


**The level of carbonylated (oxidized) proteins were decreased in mitochondrial fraction of MTS-CRBN overexpressed SH-SY5Y cells.**

The level of carbonylated (oxidized) proteins was reduced in total cellular extract of MTS-CRBN overexpressed SH-SY5Y cells (*$p < 0.05$, Fig. 4C). To examine the proteolytic activity of mitochondrially-expressed CRBN under oxidative stress conditions, we compared the level of oxidized proteins in the mitochondrial fractions from control and MTS-CRBN expressed cells. The level

of carbonylated proteins was detected by western blotting using antibody against 2,4-dinitrophenol (DNP), as performed in Fig.4C. In control cells, hydrogen peroxide treatment induced the generation of oxidized proteins in the mitochondrial fraction. Especially, the protein bands with estimated molecular mass of 60 and 68 kDa were significantly oxidized (Arrowheads). The level of these oxidized protein bands were suppressed in mitochondrial fraction of MTS-CRBN overexpressed SH-SY5Y cells compared with that of control cells.
